# Evolution of gamma knife capsulotomy for intractable obsessive-compulsive disorder

**DOI:** 10.1038/s41380-018-0054-0

**Published:** 2018-05-09

**Authors:** Euripedes C. Miguel, Antonio C. Lopes, Nicole C. R. McLaughlin, Georg Norén, André F. Gentil, Clement Hamani, Roseli G. Shavitt, Marcelo C. Batistuzzo, Edoardo F. Q. Vattimo, Miguel Canteras, Antonio De Salles, Alessandra Gorgulho, João Victor Salvajoli, Erich Talamoni Fonoff, Ian Paddick, Marcelo Q. Hoexter, Christer Lindquist, Suzanne N. Haber, Benjamin D. Greenberg, Sameer A. Sheth

**Affiliations:** 1 Department and Institute of Psychiatry, Hospital das Clinicas HCFMUSP, Faculdade de Medicina, Universidade de Sao Paulo, São Paulo, Brazil; 2 Departments of Psychiatry and Human Behavior and Neurosurgery, Warren Alpert Medical School of Brown University and Veterans Affairs Medical Center of Providence, Providence, RI USA; 3 Division of Neurosurgery, Sunnybrook Health Sciences Centre, Harquail Centre for Neuromodulation, University of Toronto, Toronto, Ontario Canada; 4 Discipline of Neurosurgery, Universidade Federal de São Paulo, São Paulo, SP Brazil; 5 Hospital do Coração, São Paulo, Brazil; 6 Department of Neurology, Hospital das Clinicas HCFMUSP, Faculdade de Medicina, Universidade de Sao Paulo, São Paulo, Brazil; 7 National Hospital for Neurology and Neurosurgery, London, UK; 8 Gamma Knife Centre, BUPA Cromwell Hospital, London, UK; 9 University of Rochester School of Medicine, Rochester, New York USA; 10 McLean Hospital, Harvard University, Boston, USA

**Keywords:** Psychiatric disorders, Neuroscience

## Abstract

For more than half a century, stereotactic neurosurgical procedures have been available to treat patients with severe, debilitating symptoms of obsessive-compulsive disorder (OCD) that have proven refractory to extensive, appropriate pharmacological, and psychological treatment. Although reliable predictors of outcome remain elusive, the establishment of narrower selection criteria for neurosurgical candidacy, together with a better understanding of the functional neuroanatomy implicated in OCD, has resulted in improved clinical efficacy for an array of ablative and non-ablative intervention techniques targeting the cingulum, internal capsule, and other limbic regions. It was against this backdrop that gamma knife capsulotomy (GKC) for OCD was developed. In this paper, we review the history of this stereotactic radiosurgical procedure, from its inception to recent advances. We perform a systematic review of the existing literature and also provide a narrative account of the evolution of the procedure, detailing how the procedure has changed over time, and has been shaped by forces of evidence and innovation. As the procedure has evolved and adverse events have decreased considerably, favorable response rates have remained attainable for approximately one-half to two-thirds of individuals treated at experienced centers. A reduction in obsessive-compulsive symptom severity may result not only from direct modulation of OCD neural pathways but also from enhanced efficacy of pharmacological and psychological therapies working in a synergistic fashion with GKC. Possible complications include frontal lobe edema and even the rare formation of delayed radionecrotic cysts. These adverse events have become much less common with new radiation dose and targeting strategies. Detailed neuropsychological assessments from recent studies suggest that cognitive function is not impaired, and in some domains may even improve following treatment. We conclude this review with discussions covering topics essential for further progress of this therapy, including suggestions for future trial design given the unique features of GKC therapy, considerations for optimizing stereotactic targeting and dose planning using biophysical models, and the use of advanced imaging techniques to understand circuitry and predict response. GKC, and in particular its modern variant, gamma ventral capsulotomy, continues to be a reliable treatment option for selected cases of otherwise highly refractory OCD.

## Introduction

Patients with obsessive-compulsive disorder (OCD) suffer from intrusive thoughts, fears, or images (obsessions), with or without ritualized repetitive behaviors (compulsions), which are carried out to reduce the anxiety or discomfort elicited by obsessions or by subjective feelings. Examples include the need to relieve a tactile sensation or achieve a “just right” feeling [1–3]. The lifetime prevalence of OCD in the general population is up to 3% [4]. The disorder is often characterized by an early age at onset, a chronic course, and a low rate of remission [5]. These factors result in high levels of economic burden, suicidality [6], and premature death [7, 8].

First-line treatments for OCD include exposure and response prevention (ERP) behavioral therapy and serotonin reuptake inhibitors (SRIs)—selective (SSRIs) and non-selective (e.g., clomipramine) [9, 10]. ERP and SRIs have been shown to be effective [11, 12], particularly in combination [13].

Obsessive-compulsive symptoms (OCS) are refractory in ~20% of cases (Table 1) [9, 12, 14, 15]. Non-response to first-line treatments should prompt review of the diagnosis and predictors of poor response [16–23].

**Table 1:** Therapeutic alternatives for treatment-resistant obsessive-compulsive disorder [9, 10, 121–123]

Treatment status	Possible strategies
1. Non-response to monotherapy with an SSRI^**a**^ or clomipramine or to BT alone^**b**^	Combine pharmacological and psychological treatments^**c**^
2. Non-response to SSRI + BT	Change SSRI^**a**,^^**d**^
	Change to clomipramine^**a**,^^**e**^
	Augmentation with atypical antipsychotic drugs or haloperidol^**c**^
	Intensive BT^**c**^
3. Non-response to second trial with SSRI	Augmentation with clomipramine^**e**^
	Augmentation with risperidone or haloperidol^**c**^
	Augmentation with other atypical antipsychotic drugs^**e**^
4. Non-response to clomipramine and to augmentation with atypical antipsychotic drugs or haloperidol	High-dose SSRI (off-label, informed consent required)^**e**^
	SSRI + clomipramine^**e**^
	Augmentation of SSRI with glutamatergic drugs (e.g., memantine^**e**^, topiramate^**e**^, lamotrigine^**e**^, and N-acetylcysteine), ondansetron^**e**^, nonsteroidal anti-inflammatory drugs^**e**^ (e.g., celecoxib), or mirtazapine monotherapy^**d**^
5. Non-response to the available treatments	Neuromodulatory treatments: rTMS^**e**^, GKC^**e**^, RF capsulotomy^**e**^, and DBS^**d**^

^a^Usually up to the maximum dose (fluoxetine, 80 mg; fluvoxamine, 300 mg; sertraline, 200 mg; paroxetine, 60 mg; citalopram, 40 mg; escitalopram, 40 mg; clomipramine, 250 mg) and for a period of at least 3 months

^b^For at least 20 h

^c^Grade A recommendation

^d^Grade C recommendation

^e^Grade B recommendation

For information on levels of evidence and grades of recommendation, please refer to the Oxford Centre of Evidence-Based Medicine at http://www.cebm.net/ocebm-levels-of-evidence [124].

SSRI selective serotonin reuptake inhibitor, BT behavior therapy, rTMS repetitive transcranial magnetic stimulation, GKC gamma knife capsulotomy, RF radiofrequency, DBS deep brain stimulation.

In some OCD patients (less than 1% of treatment-seeking individuals), the condition is severe and considered “intractable” [24]. In select cases, neurosurgery might be the only viable therapeutic option. Table 2 describes the current selection criteria for neurosurgical candidacy [25–27].

**Table 2:** Current overall selection criteria for neurosurgery for intractable obsessive-compulsive disorder

**Inclusion criteria**
•Main diagnosis of OCD (If comorbid Axis I or II disorders are present, OCD symptoms should be the most troublesome.)
•Y-BOCS OCD severity rating of 28 or higher (extremely ill) or 14 if only obsessions or only compulsions are present. In any potential candidate, OCD must be extremely time-consuming or impairing OCD
•≥5 years of severe OCD symptoms despite adequate treatment trials
•Refractoriness, as evidenced by insufficient response to the following:
–≥3 trials with an SRI (selective or not), at least one of which should be with clomipramine. All trials should have a minimum duration of 12 weeks, at the maximum tolerated dose
–≥2 augmentation strategies, such as the use of antipsychotic drugs (typical or atypical) or clomipramine, with adequate duration and dose
–≥20 h of OCD-specific BT (i.e., ERP). Participation for shorter times may be permitted if nonadherence is due to symptom severity rather than to noncompliance
•Independent confirmation of the above refractoriness criteria with previous mental health providers
•Age 18–75 years (increasing age is a relative contraindication)
•Ability to provide informed consent
•Appropriate expectations of the outcomes of surgery
**Exclusion criteria**
•Co-morbid psychiatric disorder that may interfere with treatment (e.g., severe personality disorder or psychosis)
•Clinically significant condition affecting brain function or structure
•Cognition in the low range
•Past history of head injury, with posttraumatic amnesia
•Current substance use disorder
•Recent suicide attempt or active, formed suicidal ideation

OCD obsessive-compulsive disorder, Y-BOCS Yale-Brown Obsessive-Compulsive Scale, SRI serotonin reuptake inhibitor, BT behavior therapy, ERP exposure and response prevention.

Neurosurgery has been an option for severe and highly refractory OCD since the mid-twentieth century, evolving from stereotactic radiofrequency (RF) ablation directed by ventriculography to modern magnetic resonance imaging (MRI)-guided procedures. In this article, we review ablative neurosurgical techniques that target the anterior limb of the internal capsule (ALIC; i.e., capsulotomy), focusing on gamma knife capsulotomy (GKC) and its most recent refinement, gamma ventral capsulotomy (GVC), which targets the ventral subregion within the ALIC.

We begin by outlining the historical development of stereotactic neurosurgery and providing an overview of the relevant neuroanatomy and target circuitry models. We then present a systematic review of the literature on GKC, discussing evolving dose and targeting strategies, efficacy, and adverse events. In that context, we delve into the development and subsequent use of GVC, the only ablative neurosurgery modality that has been studied in a double-blind, sham-controlled randomized trial. Finally, we conclude with discussions regarding future considerations for GVC, including patient selection criteria, biophysical calculations for optimal dose distribution, and the use of neuroimaging techniques to understand mechanisms of clinical response. Given the heterogeneous methods and measurement tools employed in this mostly open-label, retrospective literature, we do not attempt to perform a meta-analysis of the outcome data. Rather, we aim to comprehensively describe how the world experience with this procedure has shaped its evolution, and highlight topics of future research.

## History of neurosurgical procedures for OCD

**Fig. 1:**
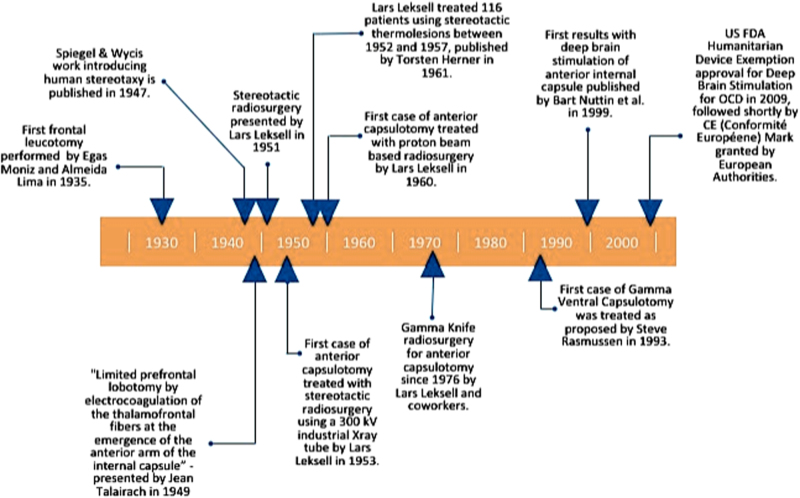
Timeline of anterior capsulotomy in the surgical treatment of obsessive-compulsive disorder [28–30, 118, 119]

**Fig. 2:**
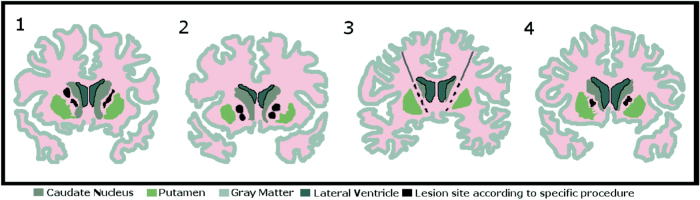
Different surgical techniques targeting the anterior limb of the internal capsule in the treatment of obsessive-compulsive disorder: **1** radiofrequency capsulotomy [113]; **2** double-shot gamma knife ventral capsulotomy [25]; **3** ventral capsular/ventral striatal deep brain stimulation [41]; and **4** magnetic resonance guided focused ultrasound at the internal capsule [40]

The development of frame-based stereotactic procedures in the late 1940s [28] enabled neurosurgical lesions to be created in a relatively precise, reproducible manner, unlike the broadly destructive frontal lobotomies that were decried for indiscriminate application and occasionally dramatic adverse effects [29]. In 1949, Jean Talairach proposed treating psychiatric disorders by using stereotactic RF thermocoagulation to create lesions in the ALIC [30]. In addition to the ALIC, targets have included the anterior cingulate cortex (ACC) and subcaudate white matter. The procedures developed were capsulotomy (targeting the ALIC) [30], anterior cingulotomy (targeting the ACC) [31–33], subcaudate tractotomy (targeting the subcaudate white matter) [34, 35], and a fourth procedure, limbic leucotomy, combining the last two [36, 37]. Figure 1 describes important landmarks and the role of anterior capsulotomy for OCD in the history of psychiatric neurosurgery [38, 39]. Modern versions of these techniques continue to be employed at specialized centers (Fig. 2) [40–42].

## Neuroanatomy of the ALIC

The ALIC carries ascending and descending fibers connecting prefrontal cortex (PFC) to deep gray matter including the thalamus and basal ganglia. These cortical areas are associated with control over emotion, motivation, and cognition, and are linked to psychiatric illnesses including OCD, major depressive disorder, schizophrenia, addiction, and several others [43–52]. Appreciation of this complex anatomy has largely been obtained from nonhuman primate tract-tracing studies [53–57]. The PFC is considerably larger and more complex in humans than in non-human primates, complicating comparisons. Methodological limitations preclude detailed studies of the anatomy and topography of the human ALIC, because neuronal tracing methods are not suitable for human use. In vivo MRI-based techniques, in particular diffusion tensor imaging (DTI), have been the mainstay of this effort.

**Fig. 3:**
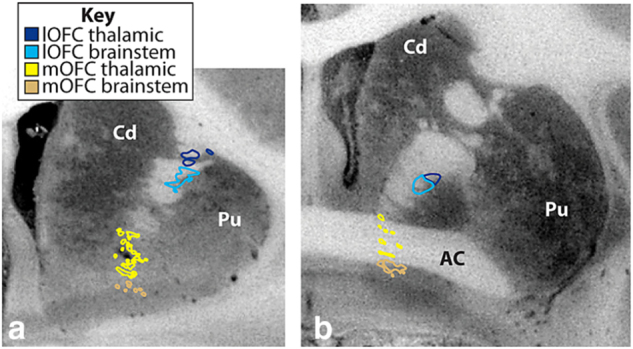
**a** and **b** Coronal sections from macaques illustrating the different positions of thalamic vs. brainstem mOFC fibers (yellow–tan) and lOFC (dark blue–light blue) entering and traveling through the IC. Brainstem fibers (tan and light blue) travel ventral to thalamic fibers (yellow and dark blue). AC Anterior commissure, Cd caudate nucleus, lOFC lateral orbital frontal cortex, mOFC medial orbital frontal cortex, Pu putamen. (Reprint with permission from, Lehman et al.) [55]

**Fig. 4:**
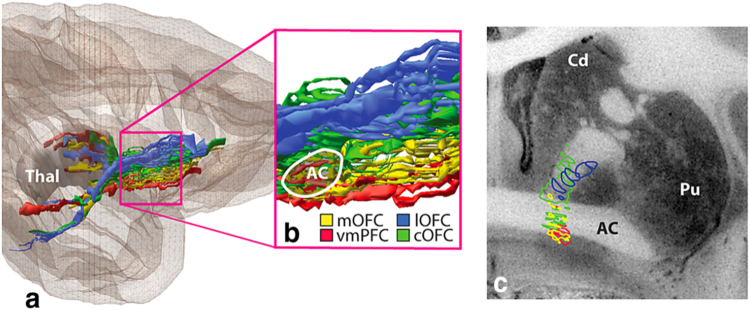
vPFC fibers through macaque internal capsule. **a** Overview of the internal capsule in the parasagittal plane. **b** Enlargement of the anterior internal capsule showing the dorsal/ventral topography rostral to the AC. Note the medial/lateral topography, with medial vPFC fibers traveling ventral to lateral vPFC axons. **c** Coronal section illustrating the organization of the vPFC fibers in the IC at the level of the anterior commissure. AC Anterior commissure, Cd caudate nucleus, cOFC central orbital frontal cortex, lOFC lateral orbital frontal cortex, mOFC medial orbital frontal cortex, Pu putamen, Thal thalamus, vmPFC ventral medial prefrontal cortex (Reprint with permission from, Lehman et al.) [55]

Frontal fibers enter the ALIC at different anterior-posterior levels and reposition themselves as they travel posteriorly [54, 58]. The nucleus accumbens/anterior commissure has traditionally been considered the ventral boundary of the ALIC [54, 59]. However, a recent study demonstrated that the small fascicules embedded within the ventral striatum contain the fibers that link medial orbital and subgenual cortex with the thalamic and brainstem fibers, thus forming the ventral continuum with the conventional ALIC [55]. Indeed, fibers from different regions of the PFC enter and travel through the ALIC in an organized manner: fibers from ventromedial regions enter the ALIC medially and ventrally, and move dorsally through the capsule to the thalamus and brainstem; fibers from ventrolateral regions arch around through the white matter to enter the ALIC more dorsally; fibers from dorsal PFC regions enter dorsally and move ventrally within the ALIC. Within the capsule, fibers from ventromedial PFC regions travel ventral to those from more lateral PFC areas (Figs. 3 and 4). The ventral component carries fibers of the ventromedial PFC, which lie ventral to those of the OFC. There are four dorsal components of the ALIC that carry fibers of vlPFC (ventrolateral ALIC), dlPFC (dorsolateral ALIC), dACC (ventromedial ALIC), and mPFC (dorsomedial ALIC) [60]. Importantly, DTI replicates this general pattern [60, 61].

## Circuitry models and neuroimaging in OCD treatment studies

Although the psychiatric neurosurgery procedures described above were developed empirically, neuroimaging, neuropsychological, and treatment studies consistently implicate these frontal-subcortical circuits in the pathophysiology of OCD [62]. Within those circuits, perfusion, or metabolism is generally greater in symptomatic OCD patients than in controls, and treatment efficacy has been strongly correlated with changes in those same circuits [63, 64]. Capsulotomy interrupts connections between prefrontal areas (dlPFC, lateral and medial OFC, vmPFC, ACC) and subcortical gray matter (ventral striatum, dorsomedial nucleus of the thalamus, hypothalamus, stria terminalis, pons, and periaqueductal gray) [26, 65]. Neuroimaging after capsulotomy for OCD has demonstrated normalized metabolism in the OFC, ACC, dorsomedial thalamus, and caudate nucleus [66]. Volumetric changes in components of this circuitry have also been observed after capsulotomy [67].

**Fig. 5:**
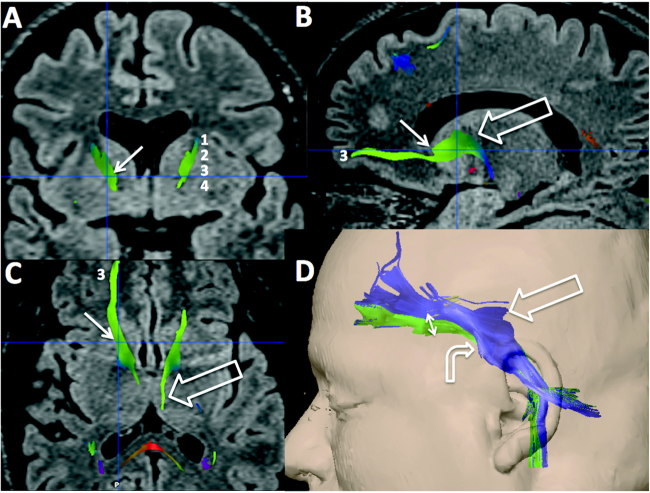
Three-dimensional reconstruction with magnetic resonance diffusion tensor imaging and tractography, showing fibers crossing the internal capsule. Coronal (**a**), sagittal (**b**) and axial (**c**) fluid-attenuated inversion recovery sequences, fused to tractography, demonstrating the relationship of the fibers of the anterior limb of the internal capsule (ALIC) passing through the capsulotomy target (thin, blue, intersecting lines, small arrows). **a** Numbers in white correspond to the order of the fibers: **1** lateral orbitofrontal cortex; **2** central orbitofrontal cortex; **3** medial orbitofrontal cortex; **4** ventromedial prefrontal cortex. **b** The large open arrow indicates connections between the cingulate, inferior orbitofrontal, central orbitofrontal, medial orbitofrontal, and ventromedial prefrontal cortex and the thalamus. **c** The fibers, numbered 3 in **a**, **b**, and **c**, are the medial orbitofrontal cortical fibers reaching the thalamus (large open arrow). **d** Three-dimensional reconstruction showing an overview of all fibers passing through the ALIC. The large open arrow indicates the medial orbitofrontal cortical fibers reaching the thalamus. The double arrow represents the fibers affected by capsulotomy. The curved arrow indicates the cingulate, inferior orbitofrontal, central orbitofrontal, medial orbitofrontal, and ventromedial prefrontal cortex connections with the brainstem (forebrain bundle), a reinforcement system important in OCD. For more information refer to Lemaire et al. [120]

The specific ALIC fiber bundles that may modulate clinical response remain to be determined. The clinical effectiveness of capsulotomy may be attributable to its effects on ventral capsule targets, affecting networks involving brain structures such as OFC and vmPFC [68, 69]. Because of significant inter-individual heterogeneity of the superior-inferior spread of capsular fibers, the optimal target location may vary between individuals [70]. In the ventral capsule (Fig. 5), lesions may need to incorporate fibers to the ventromedial caudate as well as those to the core and shell of the nucleus accumbens [64, 65].

## GKC: principles and targeting

**Fig. 6:**
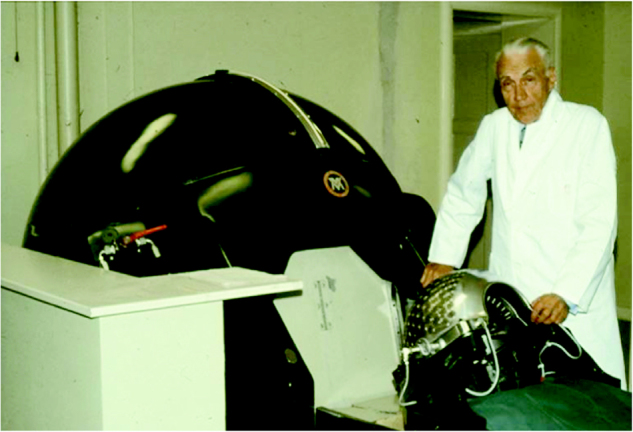
Lars Leksell and the prototype Gamma Knife installed in the Sophiahemmet Hospital in Stockholm in 1968. Photo: Georg Norén (1979)

Soon after Spiegel and Wycis demonstrated the first stereotactic procedure in humans, Swedish neurosurgeon Lars Leksell began developing a method for delivering radiation to the brain stereotactically. Rather than placing a probe inside the brain and creating a thermal lesion, he used focused ionizing radiation (X-rays) to produce the same effect without open surgery. In 1953, 4 years after the first description of RF capsulotomy, Leksell performed the first radiosurgical capsulotomy [71]. The ability to treat intracranial pathology or create functional lesions without open-skull surgery represented a major advance (Fig. 6).

In 1967, Leksell presented a radiosurgical apparatus intended for research and routine clinical use, equipped with sources of a radioactive isotope of cobalt (^60^Co) emitting high-energy gamma rays with a half-life of 5.27 years. The “gamma knife” (GK) employs many ^60^Co sources, arranged in a hemispherical or conical configuration within a helmet-like part of the device [72]. Modern GK models have 192 ^60^Co sources, and the beams from each source pass through a separate cylindrical channel, or collimator, to produce beams of 4, 8, or 16 mm width as measured at their intersection (focus). A single exposure of a target volume (often called “a shot”) produces a maximum dose at the focus of the 192 beams. The dose depends on the exposure time and the dose rate. The prescription dose is the dose given to achieve the desired biological effect (destruction of a tumor, obliteration of an arteriovenous malformation (AVM), or making a lesion in the brain for treatment of OCD). In the ideal dose plan the prescription dose conforms completely to the target volume. Several different dose plans may achieve this requirement. The plans may differ in the number of shots used, the collimators used, the exposure time, and number of beam channels used. Clinically it is of great importance that the dose distribution outside and inside the target volume may differ significantly between plans with the same prescription dose and conformity. These differences translate into differences in chance of success and risk of complications.

The first Gamma Knife capsulotomies for refractory OCD and other anxiety disorders were performed in 1976 [73, 74]. In the following systematic review we have extracted information from the literature about GKC targets, dose plans, imaging, and clinical treatment effects.

## Systematic review of GKC

### Methods

**Fig. 7:**
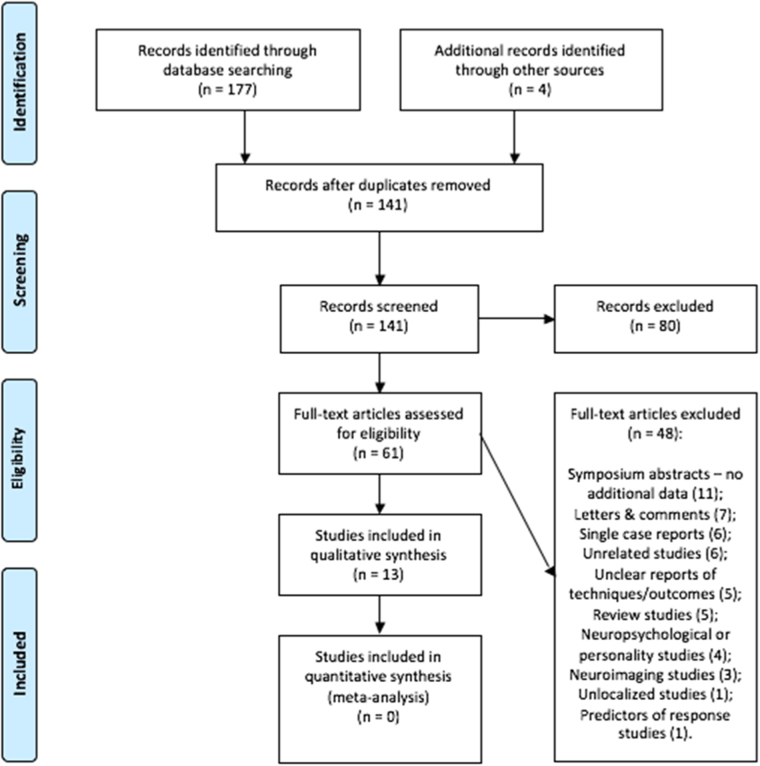
PRISMA 2009 flow diagram

We searched the main biomedical databases (PUBMED, EMBASE, and Cochrane) systematically, using terms related to GKC and OCD. See the online supplementary information for specifics. The search strategy included the following terms: (obsessive-compulsive disorder OR obsessive compulsive disorder OR OCD) AND (radiosurgery OR gamma knife OR gamma ventral OR gamma capsulotomy OR capsulotomy). Figure 7 depicts the resulting flow diagram of references identified.

### Evolution of GKC technique and targeting

Between 1976 and 1979, Leksell, Backlund, and Rylander at the  Karolinska Institute in Stockholm, Sweden treated 21 patients with anxiety disorders (12 of whom had OCD) using the first prototype of the Gamma Knife (GK I, see Fig. 6). The 179 sources of GK I were arranged in a hemispherical configuration and the beam channels were rectangular in shape (3 × 5 mm or 3 × 11 mm). The target for these treatments was the middle part of the anterior limb of the internal capsule (10 mm in front of the anterior commissure, 8 mm above the intercommissural line, and on average 17 mm lateral to the midplane). The majority of patients were treated using the 3 × 5 mm collimator and a maximum dose of 160–180 Gy [75].

Mindus and Lindquist of the  Karolinska Institute treated another cohort of OCD patients in 1985. They used the second Gamma Knife prototype (GK II), with 179 sources and 4, 8, and 14 mm cylindrical collimators, to treat two patients, and then used the Gamma Knife Model B, with 201 sources and 4, 8, 14 and 18 mm cylindrical collimators, to treat 13 patients. The cylindrical collimators of the Model B create spheroidal isocenters, rather than the rectangular shape created by the original GK unit. The treatment plans aimed to cover a region of the ALIC similar to what was covered in the previous cohort by stacking three spheroidal shots using the 4 mm collimator. One patient was treated with 160 Gy maximum dose, and nine patients with 200 Gy [63]. Due to the geometry of the source arrangement in the helmet of the Model B, the spheroidal isocenters are slightly oblate (wider around the equator than tall, like a lentil). Differences in this geometrical parameter between GK units has important implications on treatment effects, as discussed below in Future Challenges: Target Optimization.

**Fig. 8:**
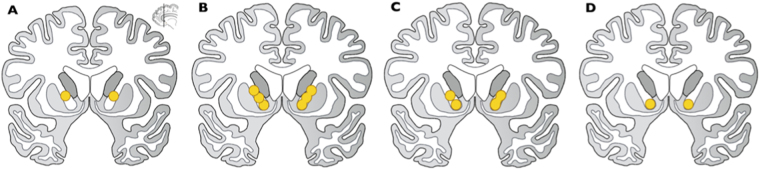
History of Gamma Knife capsulotomy for OCD: **a** original Gamma Knife capsulotomy target (GK I) and recently revisited [75, 77]; **b** triple isocenters used in early GK B series [63, 84]; **c** Gamma ventral capsulotomy (double-shot bilateral lesions, 4-mm collimators) [69, 78]; **d** single-shot Gamma ventral capsulotomy (ventral-capsule single-shot bilateral lesions, 4-mm collimators) [79]

In the United States, Rasmussen and Lindquist started the first GKC program for OCD with Mindus as an invited consultant in 1993. Fifteen patients were treated with a single 4-mm bilateral shot at 180 Gy using the GK Model U. The Model U has a different arrangement of sources, resulting in spheroidal isocenters that are slightly prolate (taller than wide, like a rugby football stood on end). For these first 15 patients, there was a single target, located centrally in the internal capsule, 1/3 of the distance up from the base of the IC. In the axial plane, the posterior part of the 20% isodose line intersected the genu of the IC (Fig. 8) [76]. Of those 15 patients, 13 underwent a second procedure, receiving another 180 Gy, 4-mm bilateral shot immediately ventral to the previous midpoint shot, bordering the ventral striatum. A refined technique combining both of the “shots” at one time (“double shot”) was subsequently used in 40 patients, 22 treated with the Model U, and 18 with the Model C [77]. The term gamma ventral capsulotomy (GVC) was coined to describe this “double-shot” procedure, with 4-mm collimators targeting the ventral ALIC and bordering the ventral striatum.

In collaboration with Butler Hospital/Brown Medical School (BH/BMS), a group at the University of São Paulo (USP), Brazil, treated five patients, reproducing all parameters of the 180 Gy double-shot GVC technique with a GK Model B [69]. Following that pilot study, the same group conducted a double-blind, sham-controlled, randomized trial involving 16 patients.

Groups at the University of Pittsburgh (UPitt) and University of Virginia (UVA) have proposed reducing the radiation dose while maintaining ventrally focused targets in the ALIC. The UPitt group treated three patients using GVC with 140–150 Gy (one using the model C, and two using the model 4C) [78]. The UVA group treated five patients with a single bilateral ventral shot (140–160 Gy), using the newer GK model Perfexion, which differs from previous models in its source geometry but with isodose distributions similar to models B and C, creating oblate spheroidal isocenters [79].

Table 3 presents the different GK models, doses, and targets selected in various GKC studies since those initial reports. Figure 8 summarizes the evolution of isocenter distribution over the years. GKC treatments initially targeted the middle part of the anterior limb of the internal capsule, further expanding to aim at the entire ALIC, and then evolving to a reduced number of isocenters until a more focused strategy targeting the ventral portion of the capsule was implemented.

**Table 3:** Gamma knife models, doses, and targets selected in various gamma knife capsulotomy studies

Study	Technique	Gamma Knife Model	No. of patients	Dose, Gy (*N* of targets)	No. of isocenters (*N*)	Dorsal-ventral extension in internal capsule	Collimators, mm (*N* of patients)	Reoperations
Rylander et al. [74], Kihlström et al. [75]	Gamma Knife capsulotomy	GK I (first prototype)	21	80 (2) 100 (2) 120 (4) 140 (4) 152(2) 160 (25) 170(1) 180(2)	1	Midcapsular	3 × 5 mm (14) 3 × 11(7)	3
Kihlström et al. [63], Lippitz et al. [83]	Gamma Knife capsulotomy	GK II (second prototype)	3	100 (1) 120(1) 160(4)	1	Midcapsular	4 mm (1) 8 mm (2)	1
Kihlström et al. [63]	Gamma Knife capsulotomy	B	11	160 (2), 200 (9)	1 (1), 3 (9), 4 (1)	Midcapsular and Dorsal, mid and ventralcapsular	4 (10), 8 (1)	1
Rasmussen et al. [125]	Gamma ventral capsulotomy, single and double-shots	U	15	180 (35)	1 (first 15 pt.), 2 (13 of the first 15 pt)	Midcapsular (first 15 pt.), mid and ventralcapsular (13)	4 (15)	13 of the first 15 pt. added a second ventral lesion
Lopes et al. [69]	Gamma ventral capsulotomy, double-shot	B	5	180	2	Mid and ventralcapsular	4 (5)	None
Kondziolka et al. [78]	Gamma ventral capsulotomy, double-shot	NA	3	140 (2), 150 (1)	2	Mid and ventralcapsular	4 (3)	None
Sheehan et al. [79]	Gamma ventral capsulotomy, single-shot	Perfexion	5	140 (3), 160 (2)	1 (ventralcapsular)	Ventralcapsular	4 (5)	None
Lopes et al. [25]	Gamma ventral capsulotomy, double-shot	B	12	4 (40)	2 (12)	Mid and ventralcapsular	4 (12)	None
Peker et al. [126]	Gamma ventral capsulotomy, single and double-shots	C (1) and Perfexion (9)	10	140, 150	1 (2), 2 (8)	NA	4 (10)	NA
Rasmussen et al. [77]	Gamma ventral capsulotomy, single and double-shots	U (37) and C (18)	55	180 (55)	1 (first 15 pt.), 2 (13 of the first 15 pt, reoperated); 2 (40 additional pt)	Mid and ventralcapsular	4 (55)	13 of the first 15 pt. added a second ventral lesion

Del Valle et al. (2006) was excluded because it employed GK capsulotomy (unknown number) + other ablative techniques - inconsistent data

Y-BOCS Yale-Brown Obsessive Compulsive Scale, CGI clinical global impression, NA not available.

### GKC outcomes: efficacy

Outcomes from GKC for OCD studies are shown in Table 4. Early studies from the Karolinska Institute reported some degree of clinical benefit in 36–56% of patients [63, 74, 80, 81]. Notably, patients with non-OCD anxiety disorders tended to respond more poorly [63]. Interpretation of those results is also hampered by the non-standardized selection criteria employed, including the lack at the time of a validated OCD symptom rating scale.

**Table 4:** Overview of studies involving gamma-knife capsulotomy for obsessive-compulsive disorder

Study	Technique	No. of patients	Diagnosis (*n*)	Mean time of FU (mo)	Pre-op Y-BOCS		12 mo post-op Y-BOCS		12 mo post-op improvement (%)		Last FU Y-BOCS		Last FU improvement (%)		Responders		Severe or permanent adverse events (*n*)	Response criteria
					Mean	SD	Mean	SD	Mean	SD	Mean	SD	Mean	SD	*n*	%		
Rylander et al. [74]	Gamma Knife capsulotomy	9	OCD (5); "chronic anxiety" (4)	5.4	NA	NA	NA	NA	NA	NA	NA	NA	NA	NA	5/9	55.5	NA	Loose response criteria
Lindquist et al. [127]	Gamma Knife capsulotomy	17	OCD and "anxiety neurosis" (17)	84 (first 7 pt.)	NA	NA	NA	NA	NA	NA	NA	NA	NA	NA	5/7	71.4	Lethargy (1)	NA
Kihlström et al. [63]	Gamma Knife capsulotomy	11	OCD (5); GAD or phobias (6)	NA	NA	NA	NA	NA	NA	NA	NA	NA	NA	NA	4/11	36.4	Severe fatigue (3), signs of frontal lobe syndrome (apathy, fatigue, loss of initiative, occasional disinhibition) (2)	Loose response criteria
Rasmussen et al. [125]	Gamma ventral capsulotomy, single and double-shots	35	OCD (35)	NA (min. 8 month, max. 4 years)	NA	NA	NA	NA	NA	NA	NA	NA	NA	NA	1/15 (single-shot), 5/13 (second, additional shot), 10/18 (double-shot)	6.7 (single-shot), 38.5 (second additional shot), 55.6 (double-shot)	Headache and cerebral edema (3/15 additional isocenter), asymptomatic caudate infarctions (2/15 additional isocenter), mania (1/15 additional isocenter); headachea and cerebral edema (3/20 double-shot), mania (2/20 double-shot), apathy and amotivation (1/20 double-shot)	At least 35% reduction in the Y-BOCS
Lippitz et al. [83]	Gamma Knife capsulotomy	10	OCD (10)	NA	NA	NA	NA	NA	NA	NA	NA	NA	74.6	42.7	7/10	70	NA	At least 50% reduction in Y-BOCS or Brief Psychiatric Rating Scale
Rück et al. [84]	Gamma Knife capsulotomy	9	OCD (9)	139. 6	33.4	4.3	17	13.9	50.3	36.3	14.3	12.1	55.8	36.3	5/9	55.6	Chronic brain edema (1), radiation necrosis with sequelae (1), cognitive changes (1), apathy (2), urinary incontinence (1), seizures (1), sexual disinhibition (1)	At least 35% reduction in the Y-BOCS
Lopes et al. [69]	Gamma ventral capsulotomy, double-shot	5	OCD (5)	48	32.2	1.48	20.2	10.4	38	31.1	20.6	12.3	36.4	37.9	3/5	60	Weight changes (4)	At least 35% reduction in the Y-BOCS + CGI scores "much iimproved" or "very much improved"
Kondziolka et al. [78]	Gamma ventral capsulotomy, double-shot	3	OCD (2); skin-picking disorder (1)	41.6	37.3	2.9	NA	NA	NA	NA	16.3	8.6	55.1	26.3	2/3	66.7	NA	NA
Rasmussen et al. [76]	Gamma ventral capsulotomy, single and double-shots	55	OCD (55)	NA (max. 20 years)	NA	NA	NA	NA	NA	NA	NA	NA	NA	NA	1/15 (single-shot), 5/13 (second, additional shot), 13/22 (double-shot)	6.7 (single-shot), 38.5 (second additional shot), 59.1 (double-shot)	Asymptomatic brain cysts (2), symptomatic brain cyst (1, with headache, dizziness, and visual changes, requiring drainage), headaches and cerebral edema (10), apathy and amotivation (1)	At least 35% reduction in the Y-BOCS
Sheehan et al. [79]	Gamma ventral capsulotomy, single-shot	5	OCD (5)	22.2	32.4	1.5	NA	NA	NA	NA	16.2	8.3	50.5	23.3	4/5	80	None described	NA
Lopes et al. [25]	Gamma ventral capsulotomy, double-shot	16	OCD (16)		34.8 (Sham group)	4	31.9 (Sham group)	4.1	7.4	13.9	NA (Sham Group)	NA	NA (Sham Group)	NA	0/8 (12 month)	0	Increased appetite and weight (4) (Sham Group)	At least 35% reduction in the Y-BOCS + CGI scores "much iimproved" or "very much improved"
				54.5 (Active group)	32.5 (Active group)	0.7	20.9	11	36.9	31.7	17.8	10	46.8	26	2/8 (12 month); 5/8 (last FU)	25 (12 month); 62.5 (last FU)	Manic episode (2), delirium + perseverations for one week (1), increased appetite and weight (6), memory deficits for 10 months (1), asymptomatic brain cyst (1), substance abuse (1) (all operated patients)	
				55.2 (all operated patients)	33.1 (all operated patients)	3.3	21.8	12.6	34.9	35.5	17.3	13.1	51.4	33.5	7/12 (last FU, all operated patients)	58.3		
Peker et al. [126]	Gamma ventral capsulotomy, single and double-shots	10	OCD (10)	9 (median)	38 (median)	NA	NA	NA	NA	NA	16 (Median)	NA	NA	NA	7	70	None described	NA
Rasmussen et al. [77]	Gamma ventral capsulotomy, single and double-shots	55	OCD (55)	36	33.3 (single-shot repeated, 15 pt)	4.8	30.7 (single-shot repeated, 15 pt)	7.6	17.8 (single-shot staged)	NA	19.3 (single-shot repeated, 15 pt)	11.3	40 (single-shot staged)	NA	1/15 (single-shot), 5/13 (single-shot repeated)	6.7 (single-shot), 38.5 (second additional shot)	Headaches (with transient edema in 5 pt), nausea, vomiting, radionecrosis (1), mania (3), insomnia, anxiety, altered mood, complaints of poor memory/concentration, lethargy, asymptomatic caudate infarcts (6), asymptomatic brain cyst (2), symptomatic brain cyst (1 – with headache, dizziness, visual changes, requiring neurosurgery)	At least 35% reduction in the Y-BOCS
					34.2 (double-shot, 40 pt)	3.2	20.3 (double-shot, 35 pt)	7.3	40.4 (double-shot)	NA	16.8 (double-shot, 32 pt)	8.3	52.7 (double-shot)	NA	10/18 (double-shot)	55.6 (double-shot)		

OCD obsessive-compulsive disorder, Y-BOCS Yale-Brown Obsessive Compulsive Scale, CGI clinical global impression, FU follow-up, GAD generalized anxiety disorder, NA not available.

The most commonly used symptom scale for OCD, the Yale-Brown Obsessive Compulsive Scale (Y-BOCS), was developed in 1989 [82]. Lippitz et al. [83] used the Y-BOCS in combination with other standardized scales to measure symptom severity and reported a ≥50% improvement in the scores in 70% of the patients undergoing GKC. The most common modern criterion for a full treatment response in OCD patients is a 35% decrease in the Y-BOCS score. Using that criterion, Rück et al. [84] of the Karolinska reported that 56% of patients undergoing GKC responded.

At BH/BMS, Rasmussen and colleagues used the same criteria to define response when they began performing GKC in 1993 [76]. In their first cohort, 15 patients were treated with a single, bilateral mid-capsule 4-mm shot, and only one individual achieved a response after a mean follow-up of 9 months [76]. There was no group improvement in the Y-BOCS score or in other measures of global improvement. Thirteen of those first 15 patients underwent a second more ventral shot. At 12 months, five (38%) of the 13 patients who underwent the completion GVC were full responders and two (15%) were partial responders (25–34% reduction in Y-BOCS score). Across the cohort, there was a significant improvement in OCD symptoms, depression, and anxiety that continued out to the year 3 follow-up. At 3 years, 7/13 (54%) were full responders, and 2/13 (15%) were partial responders. Global functioning scores also significantly improved at all time points [77].

Because of the success of including the more ventral portion of the ALIC within the lesion, the BH/BMS group subsequently treated 40 patients with double-shot GVC. At 12 months, 22/40 (55%) of the patients achieved a full response and 9/40 (23%) achieved a partial response. All of those patients subsequently maintained their improvement, and additional patients achieved a complete response after the 12-month point. At 36 months (using last observation carried forward), 30 (75%) were full responders and five (12.5%) were partial responders [77].

In their pilot GVC study, the USP group enrolled five patients [69]. A full response was defined as a Y-BOCS score decrease ≥35% and Clinical Global Impressions Improvement (CGI-I) scale [85] score of 1 (“very much improved”) or 2 (“much improved”). At 48 months, 3 (60%) were complete responders (2 after 12 months and 1 after 48 months), and 1 (20%) was a partial responder. The mean Y-BOCS score before and after the procedure (at 48 months) was 32.2 and 20.6, respectively, an overall reduction of 36%. Measures of anxiety and depression also improved in the sample as a whole. The mean Beck Depression Inventory score was 25.2 and 16.6 before and after the procedure, respectively (34% reduction), and the Beck Anxiety Inventory score was 27.6 and 12.6, respectively (54% reduction).

The UPitt study described open-label GVC treatment using a single ventral shot in 3 patients [78]. The inclusion criteria addressed severity (Y-BOCS score ≥ 24) but not comorbidities or refractoriness; nor did the authors employ formal response criteria, although pre- and post-GVC Y-BOCS scores were reported. In one patient, the score decreased from 35 to 24 (a 29% reduction) after 55 months. In another, it decreased from 39 to 32 (an 18% reduction) after 7 months, to 8 (a 79% reduction) after 17 months, to 4 (90% reduction) after 30 months, and to 7 (82% reduction) after 42 months. In the remaining patient, Y-BOCS decreased from 39 to 18 (54%) at 28 months. If the 35% reduction in Y-BOCS score response criterion were applied, 2 (67%) of the three patients would be categorized as complete responders.

**Fig. 9:**
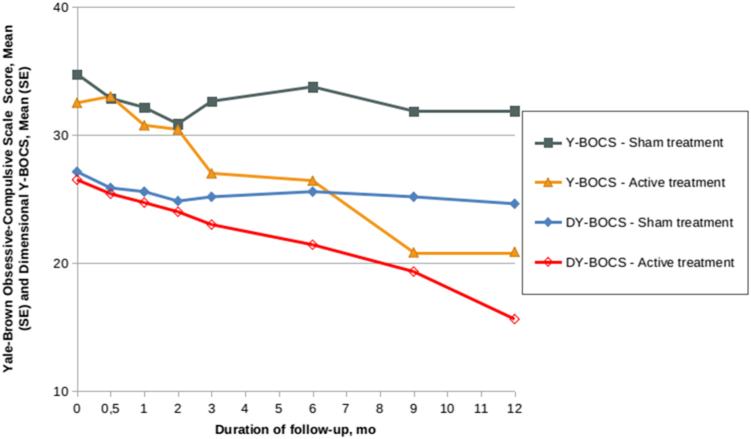
Mean (SE) Yale-Brown obsessive-compulsive scale (Y-BOCS) and mean (SE) dimensional Yale-Brown obsessive-compulsive scale (DY-BOCS) scores for the sham treatment and active treatment groups during the first 12 months of follow-up (double-blind phase). The mean Y-BOCS scores decreased 36.9% in the active treatment group and 7.4% in the sham group (*P* = 0.04988). The median DY-BOCS scores decreased 40.7% in the active group and 8.9% in the sham group (*P* = 0.01)(modified from Lopes et al.) [25]

On the basis of those promising results, the USP group conducted a double-blind, sham-controlled randomized trial involving 16 patients [25]. Eight patients were randomized to active GVC, and the other eight to a well-executed sham procedure that included the same head frame placement as the active procedure, but with a sham attachment on the GK device. The double-blind period of the study lasted 12 months. Using the same strict criteria for response used in their pilot study (change in Y-BOCS plus CGI-I), 2/8 patients randomized to GVC achieved response at 12 months, compared with 0/8 patients in the sham arm. This difference, the primary outcome measure, did not achieve statistical significance. However, median Y-BOCS scores at 12 months were 23.5 for GVC vs. 31 for sham, a statistically significant difference (*p* = 0.01). The Y-BOCS reduction over that same follow-up period was also significantly different across groups (mean 36.9% for GVC vs. 7.4% for sham, *p* = 0.04988) (Fig. 9).

Following the 12-month blinded period, there was a 54-month open-label follow-up period. During that period, three patients in the active arm who were non-responders in the blinded period became responders at months 14, 18, and 24, respectively. In addition, patients in the sham group were offered open-label active treatment, and four of eight subsequently underwent GVC. Of those four, two achieved a complete response, at months 6 and 36, respectively. Therefore, 7/12 (58%) of the patients who underwent GVC were complete responders after long-term follow-up in the open-label period of the study. The range of change in Y-BOCS score for those patients who received GVC was 0–100%, with a median of 60.0% and mean of 51.5 ± 33.5%. Among those who responded, the interval between GVC and clinical response was 6–36 months.

It is noteworthy that the criterion for complete response in this study incorporated Y-BOCS change plus change on the CGI-I score. Previous studies in the Y-BOCS era used only Y-BOCS change as a criterion for complete response (Table 4). If only the Y-BOCS criterion had been applied in the USP trial, the results would have differed: three (rather than two) of the eight GVC patients would have been categorized as complete responders in the blinded period compared to none in the sham group. Two additional patients during the open label follow-up would also have been labeled as responders, for a total of 9/12 (75%) overall.

More recently, Sheehan et al. from UVA evaluated five patients who underwent open-label GVC with a single ventral shot [79]. Patients included had an entry Y-BOCS score ≥ 24 and refractoriness but, unusually for this patient population, were judged to be without notable psychiatric comorbidities. The median pre-GVC Y-BOCS score was 32 (range, 31–34). Formal response criteria were not stated, although pre- and post-GVC Y-BOCS scores were provided. Four of the five patients (80%) were considered to have achieved “marked clinical improvement.” In those four patients, the percentage reduction in Y-BOCS scores was 59–62%, which meets the conventional ≥35% response criterion, at a median follow-up of 24 months (range 6–33 months). There were subjective indications of improvement as early as 2–3 months in three of the four responders. Although formal scales of global function were not provided, the case descriptions mention patient- and family-reported improvements in anxiety management, employment, and social function. In the single non-responder, Y-BOCS severity decreased 6%, without improvement in global function.

### GKC outcomes: adverse events and neuropsychological outcomes

In the early Karolinska cohort [63], several adverse events were observed. Of the nine patients, five (56%) exhibited severe frontal lobe edema at 12 months, with symptoms including headache, apathy, fatigue, loss of initiative, and disinhibition. Two of the patients improved over time, but three (33%) remained symptomatic.

In the later Karolinska cohort [84], patients also developed frontal lobe dysfunction, which correlated with radiation dose. Patients who received higher radiation doses from three 4-mm shots at 200 Gy or who were treated more than once were more likely to exhibit executive dysfunction, apathy, or disinhibition. One of those patients also experienced urinary incontinence and seizures. Weight gain was observed across the entire cohort, with an average gain of 6.2 kg at 12 months and 11.2 kg during long-term follow-up.

Of the 55 patients (two single shot, 53 double shot) treated at BH/BMS, three (5%) developed radionecrotic cysts 3–5 years after GVC [76]. Those three patients were part of the later cohort treated with double-shot 180 Gy GVC with the GK Model C (i.e., oblate spheroidal isocenters; see Future Challenges: Target Optimization below for detailed discussion). Two of those patients were asymptomatic, although one (2%) developed sufficient necrosis-related edema to cause headache, apathy, confusion, and other neurological changes requiring surgical decompression. This individual was left with persistent neurological sequelae. Four of 55 patients developed headaches requiring corticosteroid treatment, with resolution of symptoms. Neuropsychological assessments showed no evidence of pervasive cognitive decline in any patient. Excepting the severe adverse events associated with the necrosis-related edema, reported above, there were no significant changes in the adverse symptom profile following the procedure, as measured by the Systematic Assessment for Treatment Emergent Events (SAFTEE) [77, 86].

In the USP pilot study of 5 patients [69], one (20%) had persistent headaches for ~2 weeks that responded to oral corticosteroids, as well as weight gain. The other four patients experienced no significant adverse events.

Of the 12 patients who received GVC in the USP randomized trial, two patients with a history of hypomania experienced manic episodes that were successfully treated pharmacologically, and one patient with no history of drug abuse subsequently developed drug dependence [87]. One patient (8%) developed symptoms of delirium, confabulation, and visual hallucinations 8 months after treatment. An MRI showed peri-lesional edema, and the patient was treated with corticosteroids, with resolution of the symptoms 5 months later. Except for the severe adverse events described in one patient above, the profile of adverse symptoms described before and after surgery was similar. This was also in line with the BH/BMS findings of adverse symptoms [86].

Neuropsychological evaluations of the 12 patients from the USP trial showed no decline in cognitive or motor functions at 12 months compared to pre-procedural baseline. In fact, as a group, patients who received active GVC showed improved visuospatial memory, whereas those who received sham treatment did not [88]. Analysis of the 17 GVC patients (five from the pilot study and 12 from the RCT) [69], revealed that intellectual functioning, attention, memory, motor skills, and executive functioning improved by 12 months after GVC [88]. For details, see the Supplementary information. Likewise, no deleterious effect was found in personality of 14 of these patients assessed by standard personality instruments (the Revised NEO Personality Inventory and Cloninger’s Temperament and Character Inventory) before and 1 year after GVC. In contrast, responders had a reduction in neuroticism and an increase in extraversion, while non-responders had no changes [89].

The UPitt and UVA studies reported no adverse events [78, 79].

### GKC outcomes: summary of available evidence

The available evidence regarding GKC for severe, refractory OCD demonstrates a complex interplay between several variables, including patient selection (OCD vs. other anxiety disorders), radiation dose planning, and GK model. These variables likely contributed to the observed range in symptomatic response, adverse events, and time to clinical response. Over the decades, radiation dose has steadily decreased. In the 1980s and 1990s, patients were typically treated with three 4-mm isocenters per hemisphere at 180–200 Gy maximum dose. The Karolinska group documented response in over half of the patients receiving this dose [84]. On the other hand, edema, radiation necrosis-induced cysts, and clinical evidence of frontal lobe syndrome were observed in several patients.

At BH/BMS, investigators initially attempted to use a lower total dose, restricting the distribution to a single 4-mm isocenter at 180 Gy near the dorso-ventral mid-point of the ALIC [76]. A poor response rate prompted them to use a two-shot GVC procedure with a larger resulting lesion volume. The first GVC cohort treated at that center (two shots, staged) showed a 38% full and 15% partial response rate at 12 months with minimal morbidity. The second cohort showed a 55% full response rate at 12 months, but a small (7% mild, temporary, 2% permanent) but non-negligible rate of adverse events.

The USP pilot study and subsequent larger RCT used the same 180-Gy two-shot GVC with a GK model B. Response rate in the 12-month blinded phase was 25 or 37.5% (depending on response criterion employed, as discussed above), and 58 or 75% during the long-term (mean 4.5 years) open label follow-up. These data underscore the importance of appreciation of the variable and potentially long (months to >1 year) interval between treatment and response. The reduction in number and dose of the isocenters also likely contributed to the observed reduction in adverse events, with only one patient (6% if considering the entire sample of 17 patients) developing temporary signs of frontal dysfunction. Moreover, in the group as a whole, detailed neuropsychological and personality assessments demonstrated overall improvement in several cognitive functions and no impairment in personality.

The UPitt and UVA studies both continued the trend of lowering radiation dose-volume delivery, with promising results (67–80% Y-BOCS response rate) without reported adverse effects in a follow-up period ranging from 6 to 60 months.

The time course of clinical response to GK capsulotomy varies significantly across individuals. Although some studies have reported symptom improvement as soon as 3 months after the procedure [79], most have reported changes in the 6–12-month range, although some patients do not improve until month 24 or even month 36 [25]. It stands to reason that decreasing the radiation dose-volume delivery may have an effect on time to response, but the available data are not granular enough to determine whether such a trend is evident.

## Future challenges

### Selection and outcome criteria

Studies over the past decades have used different entry criteria for treating OCD patients with neurosurgical interventions, and have also used different response criteria for evaluating outcomes. Table 4 shows the variation in outcome measures among different capsulotomy studies. Consistent selection and outcome criteria in future studies will facilitate comparisons and replication. Selection criteria for neurosurgery for OCD (Table 2) have been suggested in recent guidelines [90]. Additional general criteria for the pre-surgical evaluation of psychiatric patients, as well as ethical considerations, were recently described in a multi-national consensus statement [91].

An international expert panel reviewing outcome criteria for clinical trials in OCD defined clinically meaningful improvement as a ≥35% reduction in the Y-BOCS score, combined with a CGI-I score of “much improved” or “very much improved” [92]. We agree that a more holistic measurement of outcome, one that considers functioning and quality of life as well as severity of core symptoms, should be the goal for future studies in the field. The USP RCT described above [25] provides a good example of occasional patients whose Y-BOCS score improves, but who have not improved much in their day-to-day functioning. We propose that true “response” should include not only symptom scales, but also measure of global function and quality of life [93, 94].

### Sham effects: placebo and lessebo

As in any interventional study, the placebo effect can be powerful [95]. To date, only one GKC study has attempted to include a sham arm with double blinding to account for the placebo effect and observational bias [25]. It is noteworthy that none of the patients of the placebo arm of the USP RCT improved after 1 year. It is possible that the placebo effect accounts for some of the early (<6 month) improvement seen in some open-label, retrospective studies.

**Fig. 10:**
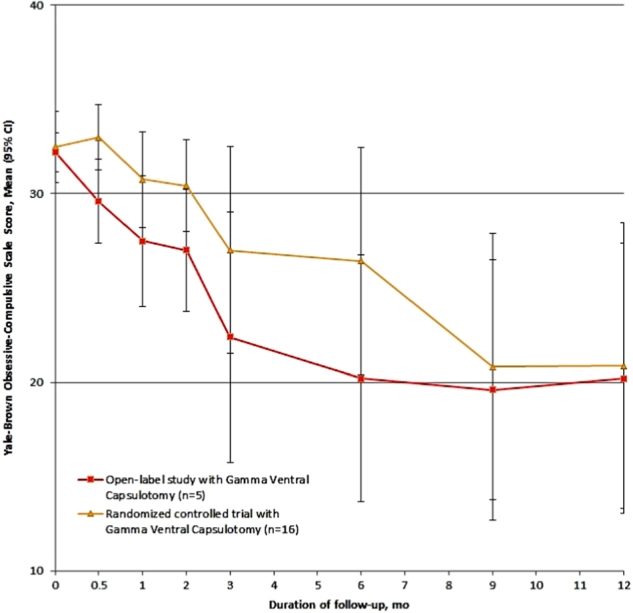
Differences in the average onset of symptom improvement, measured by the Yale-Brown obsessive-compulsive scale score, in an open-label trial of Gamma ventral capsulotomy [69] and a randomized sham-controlled trial of Gamma ventral capsulotomy [25] (here depicted only patients in the active group, N=8), both using the same technique, selection criteria, and evaluation methods.

Less often discussed but also possibly influential is the lessebo effect, i.e., that a patient entering a trial with a placebo/sham arm may experience a negative expectation of benefit when facing the concrete possibility of being given a placebo or receiving sham treatment rather than the active treatment under investigation [96]. Figure 10 illustrates this phenomenon using data from the USP open-label pilot study [69] and randomized sham-controlled trial [25]. Despite the fact that the candidacy evaluation strategy, intervention, treating team, and other variables were identical in both studies, the response occurred later in the sham-controlled trial than in the open-label pilot study. That effect may be observed in neurosurgical interventions for psychiatric disorders in general [96]. We therefore recommend that future studies, particularly those involving a placebo arm, use a longer interval for response determination.

### Clinical predictors of outcome

The search for clinical treatment predictors has been defied by the heterogeneity in the phenotypic expression of OCD [97]. Despite the challenge, symptom dimension strategies have shown some promise in establishing predictive correlations of response to pharmacological and psychological treatments [19]. For instance, patients with prominent hoarding symptoms have shown a poorer response to conventional treatment [19, 98]. Similarly, hoarding has been associated with a worse treatment response to capsulotomy [99], including GVC [100]. OCD patients with primarily hoarding symptoms tend to be excluded from surgical trials, a distinction reflected in the classification of compulsive hoarding as a separate disorder in DSM-5. Higher symmetry scores have also been associated with lack of positive response to RF capsulotomy and GKC [99], as well as to DBS [101].

### Neuroimaging predictors of outcome

The search for predictors of outcome includes those that can be identified with neuroimaging. One recent study attempted to identify preoperative neuroimaging biomarkers of response in patients undergoing RF cingulotomy for OCD, using voxel-based morphometry to study gray matter structure, and DTI to study white matter connectivity in preoperative scans [102]. A statistical model comparing responders and non-responders to cingulotomy identified cortical and connectivity differences between groups, suggesting that patient brain structure influences the likelihood of response. Future studies should apply this type of analysis to patients undergoing other surgical procedures, including capsulotomy. Such efforts should aim to provide tailored treatments, with patient-specific neuroimaging characteristics or OCS dimensions correlating with distinct neurosurgical techniques, thus optimizing clinical efficacy.

### Target optimization

**Fig. 11:**
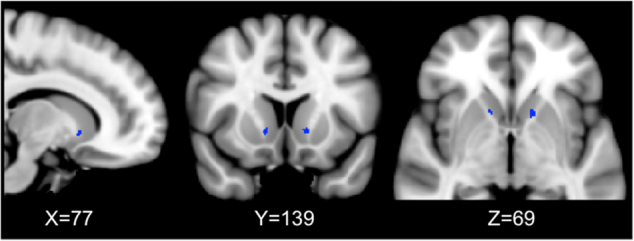
Capsulotomy lesion associated with clinical response. Post-procedural imaging data were analyzed from 26 OCD patients who had undergone GKC at BH/BMS and the University of São Paulo. The investigators used a statistical model to determine the relationship between lesion location and clinical response. The blue voxels denote regions of the lesioned area that were significantly associated with reduction in Y-BOCS score. Results are superimposed on the MNI152 non-linear 6th generation atlas

Optimizing the target involves strategies to improve efficacy and diminish side effects. One attempt to find optimal GVC lesion placement regarding treatment response used a retrospective analysis of post-operative MRI data of 26 patients undergoing GVC at USP [25, 69] (*N* = 14) and at BH/BMS [64, 76] (*N* = 12). The authors manually contoured the lesion volume on the most recent follow-up volumetric MRI scan, using two trained raters with expert supervision and confirmed inter-rater reliability measures [103]. The masked volumes were transformed to standardized image space to compare across individuals. The authors used a statistical model to determine the relationship between lesion location and clinical outcome. They found bilateral clusters of voxels in the ventral portion of the ALIC (in the coronal plane), approximately near the posterior putaminal border (in the axial plane) that were statistically related to responder status, suggesting that lesions including this region are more likely to produce a clinical response (Fig. 11) [103].

**Fig. 12:**
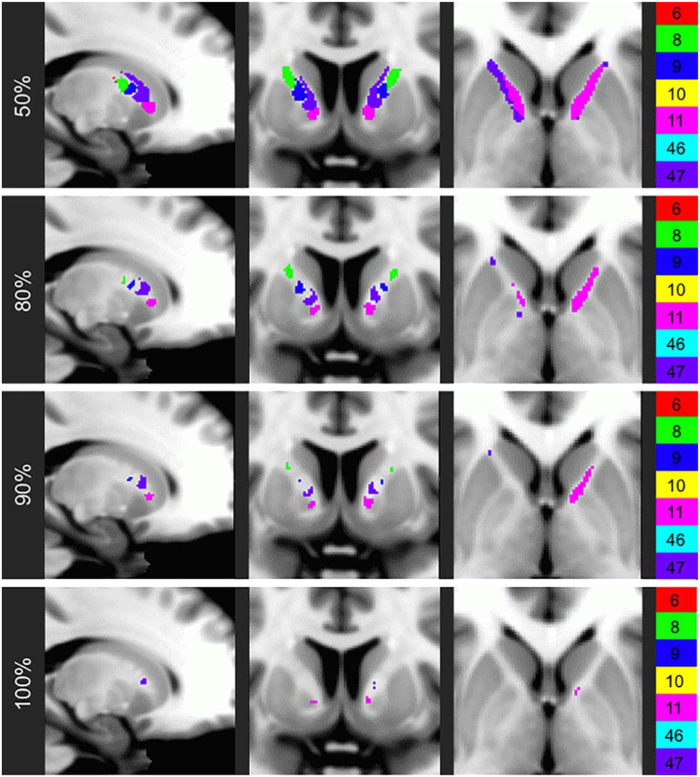
Arrangement of thalamo-prefrontal fibers within the ALIC. Probabilistic tractography analysis was performed on imaging data from 40 Human Connectome Project (HCP) subjects. Fibers were identified and color-coded based on the Brodmann Area (numbers along right margin) in which they terminated. The resulting parcellations were then thresholded at 50, 80, 90, and 100% to indicate anatomical consistency of fibers in each voxel. The dropout of voxels at higher thresholds indicates the large degree of inter-individual variability in the ALIC. (Nanda et al. with copyright permission)

To understand how interventions in this region of the ALIC produce a clinical response, it is important to understand the brain regions affected by lesions in this area. The white matter fibers within the ALIC connect prefrontal cortical regions to deep nuclei, including the basal ganglia and thalamus. As mentioned above, monkey studies have demonstrated a corticotopic organizational pattern of these fibers, with various cortical areas sending fibers through specific regions of the capsule. To study this pattern in humans, Nanda et al. [104] applied tractography analysis to diffusion tensor imaging (DTI) data from the publicly available Human Connectome Project database. They found that fibers follow a consistent gradient, with ventro-medial PFC and medial OFC fibers lying antero-ventrally, and dorsolateral PFC and supplementary motor area fibers lying postero-dorsally (Fig. 12). This organizational pattern therefore suggests that the lesion area associated with response (Fig. 11) influences connections between ventro-medial PFC and medial OFC and deep nuclei, and that modulation of activity in these cortical regions is critical to symptom response. Also evident from this analysis was the significant degree of inter-individual variability in the exact location of these thalamo-frontal fibers. Future prospective work can test the hypothesis that identifying and targeting these specific fiber bundles within individuals will result in improved outcomes. This approach has proven successful in DBS for depression [105].

**Fig. 13:**
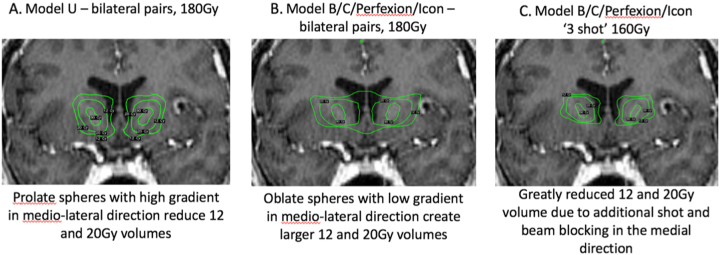
Coronal MRIs demonstrating the effect of different GK models and radiosurgical plans on radiation isodose distribution. **a** The Model U GK unit produces prolate spheroidal isocenters. The isodose lines show the volumes enclosed at 12, 20, and 90 Gy using bilateral 4-mm double-shots, with a dose of 180 Gy. The prolate geometry produces a high gradient in the medial-lateral direction such that the 12 Gy isodose line does not cross the midline. **b** The Models B, C, Perfexion, and Icon produce oblate spheroidals. When stacked in the same bilateral double-shot manner as in **a** the oblate geometry produces more medial-lateral radiation spread, resulting in larger volumes at the same isodose contours (12, 20, 90 Gy). In particular, the 12 Gy lines cross the midline, producing a particularly large 12 Gy volume. **c** By using a different radiosurgical planning strategy (a third shot, beam blocking, and lower dose of 160 Gy), the resulting isodose distribution can recapitulate that in **a** despite the fact that the geometry of the individual isocenters is still oblate. The resulting radiation volumes are again smaller, and the 12 Gy isodose lines do not cross the midline

Beyond strategies to improve likelihood of response, other strategies have focused on reducing the likelihood of side effects. As mentioned above, the most worrisome side effect of GVC is the emergence of late radionecrotic cysts and resulting cerebral edema [25, 69, 78, 106]. Such cysts were reported only in patients in whom a GK Model B or C was used, and not in patients treated with the Model U. The older Model U delivers prolate spheroidal isocenters, whereas the newer models (B, C, 4C, as well as the even newer Perfexion and Icon) deliver oblate spheroidal isocenters (Fig. 13) [107, 108]. These changes have little to no effect at the doses typically used (20–40 Gy max dose) for the most common indications for GK (brain tumors, vascular malformations, etc.). But at the much higher doses needed for GKC (140–180 Gy max dose), this difference may be of significant importance.

The oblate spheroidal geometry results in radiation isodose lines that cover a greater volume in the axial plane. Because the lesions are bilateral, the summated radiation delivery between the lesions in the medial-lateral direction, is higher with this geometry. The prolate spheroidal geometry creates isodose lines that are taller than wide, resulting in less summation across the midline. Thus the volume of tissue receiving radiation doses in the 10–40 Gy range is higher with the oblate than prolate geometry (Fig. 13) [109]. This difference is hypothesized to account for the higher likelihood of edema/cyst development in patients treated with higher prescription doses and oblate geometry.

Because all newer models of the GK produce the oblate geometry, recent efforts have focused on developing dose distribution strategies that recreate a prolate shape. Paddick and colleagues [109] have developed a dose distribution that accomplishes this goal (the “Triple Shot”). By placing an additional intermediate, lower weighted shot between the ventral and dorsal pair, and using beam blocking, the resultant dose distribution follows much more closely the pattern of the prolate double shot plan of the Model U (Fig. 13). In particular, the volumes receiving 10–40 Gy are significantly lower than in the double shot plans of the newer models, again recapitulating the dose-volume distributions of the Model U. Future studies using this dosing strategy will reveal whether this approach is successful at improving response rate while keeping a low side effect profile.

### Long-term follow-up after GKC

Complete elimination of OCS is not to be expected after surgery, and this expectation should be clearly explained to all involved. A more realistic goal is to aim for levels of improvement that enhance the effects of conventional therapies, engendering possible synergistic effects between surgical and nonsurgical treatments [110]. Therefore, pharmacological and psychotherapeutic regimens are always maintained after GVC, being reduced only when clinical improvement occurs and persists. Medications are rarely discontinued after surgery. Given the possibility of delayed side effects (e.g., swelling or cyst formation), patients should be followed for years.

A recent report from the Karolinska group took advantage of the national health registry system in Sweden to provide very long-term follow-up information (from 13 to 43 years) on 70 patients who had undergone capsulotomy [111]. A notable finding was that among the patients who were still alive, 75% were still being prescribed at least two psychiatric medications, most commonly antidepressants. An important limitation of this type of registry-based study was that finer-grain information for individual patients such as symptomatic outcome, adverse events, and details of the GKC procedure that each patient had undergone were not available. But the general findings again support the idea that severe OCD is a challenging, chronic disorder, and that even after GKC most patients still require ongoing psychiatric care [112].

### Comparison with other ALIC-focused treatments

Beyond GKC, several other neurosurgical treatment options for severe, refractory OCD are available. Examples include other capsulotomy techniques: radiofrequency (RF) [113], the original method, as well as new methods such as MRI-guided focused ultrasound (MRgFUS) [40], and laser interstitial thermal therapy (LITT) [114]. As mentioned above, other ablative options include cingulotomy [27, 31–33], subcaudate tractotomy [34, 35], and limbic leucotomy [36, 37]. Finally, DBS is available as a non-ablative, stimulation-based option [41, 42, 115].

A detailed comparison of these other techniques to GKC is beyond the scope of this review, and other such comparisons are already available in the literature. For example, Brown et al. performed a systematic review of capsulotomy (RF and GK) and cingulotomy [116], and Pepper et al. performed a comparison of capsulotomy to ALIC-focused DBS [117]. DBS has the attractive characteristics of reversibility and adjustability, but also carries intrinsic limitations given the need for expert local long-term follow-up for parameter adjustments, potential device replacements, or other surgical revisions, and other factors. MRgFUS and LITT capsulotomy are still at the proof-of-concept stage. Therefore, unless future studies with direct comparisons can demonstrate that one procedure is clearly superior in terms of efficacy and safety, it is likely that different neurosurgical techniques for psychiatric disorders will continue to coexist, with usage being determined by patient profiles, institutional availability, and experience.

## Conclusions

This review provides an in-depth account of the evolution of GK capsulotomy over the half-century since its inception. A number of “input” factors have changed over this period of time, including how patients are selected, how the treatment is administered, and how outcomes are assessed. This complex interplay of factors has in turn affected the observed “outputs,” especially symptom response and adverse event rate.

Looking at the broad sweep of the history of this procedure from the perspective of this review brings to focus two major trends. The first is the progressive reduction in radiation dose employed by studies in the past three decades since outcomes have been more consistently measured. This reduction has been accompanied by a sharp decline in adverse event rates. Whereas over half the patients in the early cohorts experienced significant neurological adverse effects at least temporarily, this rate has dropped close to zero or in some cases actually to zero in recent series.

A second important trend is the incorporation of more sophisticated analysis techniques to learn from previous experience and inform future efforts. The first few decades of GKC studies largely relied on empiric observations regarding the relationship between radiation dose strategy and the development of beneficial and adverse effects. The last several years have witnessed a more analytical approach, in which biophysical methods have led to the understanding of adverse event occurrences and even to the emergence of rationally-designed dosing strategies. Statistical models, in conjunction with collaborative efforts incorporating multi-institutional data, have started informing our targeting strategy by demonstrating relationships between lesion location and symptomatic response. More sophisticated understanding of the underlying neuroanatomy has identified the brain regions influenced by these procedures and will likely lead to individualized, patient-specific treatments in the near future.

Radiosurgical capsulotomy remains an important option in our armamentarium for treating patients with severe, refractory OCD. The therapeutic window continues to expand as outcomes improve and adverse events decline, fueled by experience and increasingly sophisticated analysis. Future efforts will focus on identifying predictors of response and tailoring treatments to individual symptoms and nuances in patient-specific neurocircuitry. Because of the highly technical and sensitive nature of these procedures, however, they should continue to remain within the purview of experienced centers capable of providing expert, multi-disciplinary, long-term care, and support for patients and families.

## Electronic supplementary material

Supplementary Information
