# Supplementary material for: Evolution of gamma knife capsulotomy for intractable obsessive-compulsive disorder
Source: Mol Psychiatry. 2018 May 9;24(2):218–40. doi: 10.1038/s41380-018-0054-0 (PMC6698394; doi:10.1038/s41380-018-0054-0)
Supplement: Supplementary file 1 — Supplementary Information [file 41380_2018_54_MOESM1_ESM.docx]

**SUPPLEMENTARY INFORMATION**

**Evolution of Gamma Knife Capsulotomy for Intractable Obsessive-Compulsive Disorder**

# **Authors**

Euripedes C. Miguel, MD, PhD, Antonio C. Lopes, MD, PhD, Nicole C. R. McLaughlin, PhD, Georg Norén, MD, PhD, André F. Gentil, MD, PhD, Clement Hamani, MD, PhD, Roseli G. Shavitt, MD, PhD, Marcelo C. Batistuzzo, PhD, Edoardo F. Q. Vattimo, MD, Miguel Canteras, MD, Antonio De Salles, MD, PhD, Alessandra Gorgulho MD, MSc, João Vitcor Salvajoli , MD, PhD., Erich Talamoni Fonoff, MD, PhD, Ian Paddick, Marcelo Q. Hoexter, MD, PhD, Christer Lindquist, MD, PhD, Suzanne N. Haber, PhD, Benjamin D. Greenberg, MD, PhD Sameer A. Sheth MD, PhD.

**Author Affiliations:** Department and Institute of Psychiatry (Drs. Miguel, Lopes, Gentil, Shavitt, Batistuzzo, Vattimo and Hoexter) and Department of Neurology (Dr. Fonoff) University of São Paulo (USP) School of Medicine, São Paulo, Brazil; the Department of Psychiatry and Human Behavior and the Department of Neurosurgery, The Warren Alpert Medical School of Brown University, Providence, RI (Drs. McLaughlin, Greenberg and Norén); Division of Neurosurgery, Toronto Western Hospital, University of Toronto, Centre for Addiction and Mental Health, Toronto, Ontario, Canada (Dr. Hamani); Hospital do Coração, São Paulo, Brazil (Drs. Salles and Gorgulho); Queen Square, London, UK (Ian Paddick); Department of Pharmacology and Physiology, Gamma-Knife Center at BUPA Cromwell Hospital, London, UK (Dr. Lindquist); Department of Pharmacology and Physiology, University of Rochester School of Medicine, New Hampshire, USA (Dr. Haber); Department of Neurosurgery, Columbia University, New York, NY, USA.

**Corresponding author:** Euripedes C. Miguel, MD, PhD, Department and Institute of Psychiatry, University of São Paulo School of Medicine. R. Dr. Ovídio Pires de Campos, 785, São Paulo, SP, 01060-970, Brazil. ([ecmiguel@usp.br](mailto:ecmiguel@usp.br))

1. **Systematic review of GK capsulotomy: Methods**

We performed a systematic search of the main biomedical databases (PubMed, EMBASE, Cochrane Library), using search terms related to radiosurgery, gamma capsulotomy and OCD. The studies were selected by two independent reviewers. Disagreements between the reviewers in terms of the selected studies were resolved by consensus. In this review, we included only original case series (minimum of two patients) or randomized trials, describing the efficacy and safety of radiosurgical procedures for OCD. Symposium abstracts were selected only if they added new, original data not described elsewhere.

As of December 2017, the search retrieved a total of 181 articles, 141 remaining after duplicates had been removed. After reading the titles and the abstracts of the remaining articles, we excluded another 80 (most because they dealt with other surgical techniques, or review studies or animal studies). We evaluated the full texts of the remaining 61 studies (Figure 7). Finally, after the exclusion of symposium abstracts, redundant studies, articles with poorly defined methods/results and papers not aimed at describing efficacy or safety issues, 13 case series and one double-blind, randomized controlled trial of GKC for OCD were eligible for this review. Of note, three studies^1-3^ not originally retrieved in the electronic search were added to the list of eligible studies, because they were cited in the selected articles and described relevant information regarding gamma knife capsulotomy for OCD. One in press study was also added. One study could not be retrieved.^4^

Because the vast majority of studies included in this review were case series with small numbers of patients and different outcome measures, we opted not to conduct a meta-analysis.

**b) Neuropsychological findings of anterior capsulotomy**

Data regarding neuropsychological outcomes of anterior capsulotomy after long-term follow-up are scarce and difficult to compare across studies, given the difference in lesion size, location, and technique (i.e., GKC vs. RF). Behavioral deficits have been described in the main text. Regarding the neuropsychological effects of capsulotomy, Nyman *et al.^5^* reported stability in cognitive functions at 7.6 years after RF surgery in 21 OCD patients, whereas others^6,7^ reported persistent mild deficits in executive functions after 7 and 11 years, respectively. One of the main concerns of those authors was the non-standardization of procedures: some patients were submitted to a second procedure and the lesion location varied among subjects.

A recent controlled study of GVC evaluated 17 patients with a comprehensive neuropsychological battery that assessed multi-domain cognitive tests. The results revealed the absence of negative changes in cognitive functioning, and, in fact, improvements in various domains, including intellectual and executive functioning, as well as attention, visuospatial memory, and motor skills, were observed 12 months after the procedure.^8^ Moreover, results from the randomized controlled trial indicated that visuospatial memory improved in the active group and showed no changes in the sham group. Therefore, the amelioration effects observed 12 months after surgery were not due to a learning effect (Figure S1). In uncontrolled studies, cognitive improvement has been reported after RF capsulotomy for OCD,^9, 10^ and that may result from direct enhancing effects on neuronal networks of operated patients or could be secondary to the amelioration of psychiatric symptoms (i.e., anxiety, OCS, and depression). Similarly, a preliminary analysis of 34 patients from Brown Medical School showed improvements in confrontation naming, tactile perception/motor speed, nonverbal memory, and verbal short delay recall improved at a mean of 8.7 years after GVC, with no worsening in any cognitive domain.^11^ Eight patients from this same sample, at a mean of 5.25 years after GVC, showed continued improvements in intellectual functioning, confrontation naming, areas of executive functioning, and visuospatial memory.^12^

In general, the literature indicates that there is no decline in cognitive functioning in the short- and long-term follow-up of OCD patients. Single or bilateral lesions in ventral anterior capsulotomy appear to be related to improvements in the cognitive functions, whereas larger and recurrent lesions appear to be related to behavioral impairments. Future studies should evaluate larger samples, for longer periods, and should use comprehensive neuropsychological batteries to elucidate the cognitive improvements reported.


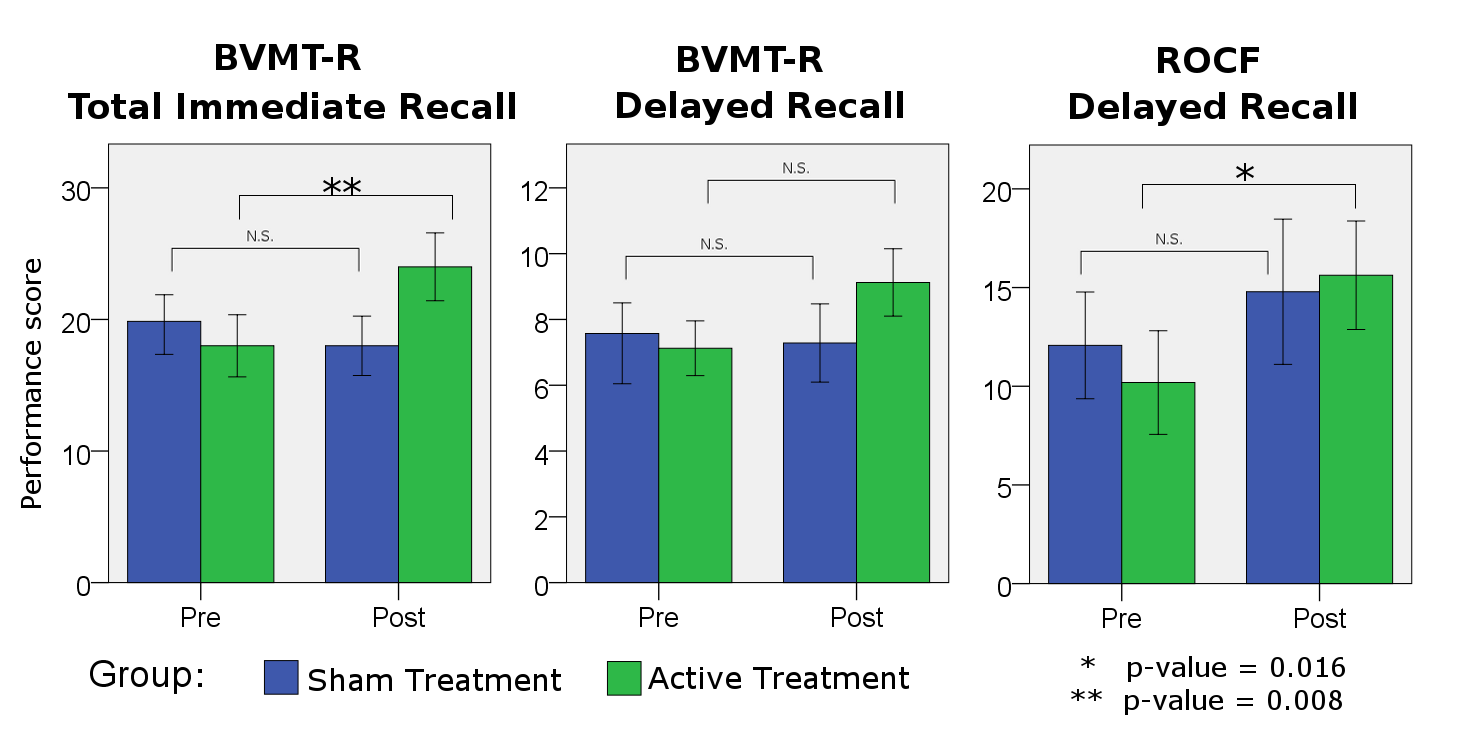


**Supplementary Figure 1.** Bar graphs showing neuropsychological improvement after GVC in two independent visuospatial tasks in a randomized controlled trial. The active group (green) showed better performance at 12 months after GVC, whereas performance in the sham-treatment group (blue) remained stable. Error bars represent 95% confidence intervals. BVMT-R: Brief Visuospatial Memory Test-Revised; ROCF: Rey-Osterrieth Complex Figure. This figure was adapted from Batistuzzo *et al.^8^*

**SUPPLEMENTARY REFERENCES**

1. Lindquist C, Kihlstrom L, Hellstrand E. Functional neurosurgery - A future for the Gamma Knife? *Stereotact Funct Neurosurg* 1991; **57**: 72–81.

2. Kihlström L, Guo W-Y, Lindquist C, Mindus P. Radiobiology of radiosurgery for refractory anxiety disorders. Neurosurgery 1995;**36**(2):294-302.

3. Rasmussen S, Mindus P, Noren G, Jenike M, Boen L, Lindquist C. Towards a double blind trial of anterion capsulotomy in obsessive compulsive disorder conference abstract. *R Coll Psychiatr Winter Meet Cardiff, Wales 21-24 January 1997.*

4. Mindus, Jenike MA. Neurosurgical treatment of malignant obsessive compulsive disorder. *Psychiatr Clin North Am* 1992; **15**: 921–938.

5. Nyman H, Andreewitch S, Lundback E, Mindus P. Executive and cognitive functions in patients with extreme obsessive-compulsive disorder treated by capsulotomy. Appl Neuropsychol 2001;**8**(2):91-98.

6. Nyman H, Mindus P. Neuropsychological correlates of intractable anxiety disorder before and after capsulotomy. Acta Psychiatr Scand 1995;**91**(1):23-31.

7. Rück C, Karlsson A, Steele JD, Edman G, Meyerson BA, Ericson K, et al. Capsulotomy for obsessive-compulsive disorder: long-term follow-up of 25 patients. Arch Gen Psychiatry 2008;**65**(8):914-921.

8. Batistuzzo MC, Hoexter MQ, Taub A, Gentil AF, Cesar RCC, Joaquim MA, *et al*. Visuospatial Memory Improvement after Gamma Ventral Capsulotomy in Treatment Refractory Obsessive–Compulsive Disorder Patients. Neuropsychopharmacology 2015;**40**(8):1837-1845.

9. Csigo K, Harsanyi A, Demeter G, Rajkai C, Nemeth A, Racsmany M. Long-term follow-up of patients with obsessive–compulsive disorder treated by anterior capsulotomy: A neuropsychological study. J Affect Disord 2010;**126**(1):198-205.

10. Taub A, Lopes AC, Fuentes D, D'Alcante CC, de Mathis ME, Canteras MM, et al. Neuropsychological outcome of ventral capsular/ventral striatal gamma capsulotomy for refractory obsessive-compulsive disorder: a pilot study. J Neuropsychiatry Clin Neurosci 2009;**21**(4):393-397.

11. McLaughlin NCR, Malloy P, Marsland R, Noren G, Greenberg B, Rasmussen S. Initial Follow-Up of Gamma Knife Ventral Capsulotomy for Treatment of Obsessive-Compulsive Disorder*.*Presented at the American Association of Anxiety Disorders in Baltimore, MD, USA, 2011.

12. McLaughlin NCR, Malloy P, Marsland R, Noren G, Rasmussen S, Greenberg B. Five Year Follow-Up of Gamma Knife Ventral Capsulotomy for Treatment Resistant Obsessive Compulsive Disorder: Preliminary Results.  Presented at the International Neuropsychological Society in Atlanta, GA, USA, 2009.
